# The promoter and transcribed regions of the *Leishmania tarentolae *spliced leader RNA gene array are devoid of nucleosomes

**DOI:** 10.1186/1471-2180-7-44

**Published:** 2007-05-22

**Authors:** Robert A Hitchcock, Sean Thomas, David A Campbell, Nancy R Sturm

**Affiliations:** 1 Molecular Biology Institute, University of California at Los Angeles, 4825 Molecular Sciences Building, 609 Charles E. Young Drive, Los Angeles, California 90095-1489, USA; 2 Department of Microbiology, Immunology, & Molecular Genetics, David Geffen School of Medicine, University of California at Los Angeles, 4825 Molecular Sciences Building, 609 Charles E. Young Drive, Los Angeles, California 90095-1489, USA; 3 Seattle Biomedical Research Institute, 307 Westlake Avenue N., Seattle, WA 98109-5219, USA

## Abstract

**Background:**

The spliced leader (SL) RNA provides the 5' m^7^G cap and first 39 nt for all nuclear mRNAs in kinetoplastids. This small nuclear RNA is transcribed by RNA polymerase II from individual promoters. In *Leishmania tarentolae *the SL RNA genes reside in two multi-copy tandem arrays designated *MINA *and *MINB*. The transcript accumulation from the SL promoter on the drug-selected, episomal SL RNA gene cassette pX-tSL is ~10% that of the genomic array in uncloned *L. tarentolae *transfectants. This disparity is neither sequence- nor copy-number related, and thus may be due to interference of SL promoter function by epigenetic factors. To explore these possibilities we examined the nucleoplasmic localization of the SL RNA genes as well as their nucleosomal architecture.

**Results:**

The genomic SL RNA genes and the episome did not co-localize within the nucleus. Each genomic repeat contains one nucleosome regularly positioned within the non-transcribed intergenic region. The 363-bp *MINA *array was resistant to micrococcal nuclease digestion between the -258 and -72 positions relative to the transcription start point due to nucleosome association, leaving the promoter elements and the entire transcribed region exposed for protein interactions. A pattern of ~164-bp protected segments was observed, corresponding to the amount of DNA typically bound by a nucleosome. By contrast, nucleosomes on the pX-tSL episome were randomly distributed over the episomal SL cassette, reducing transcription factor access to the episomal promoter by approximately 74%. Cloning of the episome transfectants revealed a range of transcriptional activities, implicating a mechanism of epigenetic heredity.

**Conclusion:**

The disorganized nucleosomes on the pX episome are in a permissive conformation for transcription of the SL RNA cassette approximately 25% of the time within a given parasite. Nucleosome interference is likely the major factor in the apparent transcriptional repression of the SL RNA gene cassette. Coupled with the requirement for run-around transcription that drives expression of the selectable drug marker, transcription of the episomal SL may be reduced even further due to sub-optimal nucleoplasmic localization and initiation complex disruption.

## Background

The spliced leader (SL) RNA gene represents the best-characterized transcriptional system in kinetoplastids. The SL RNA is a non-coding small nuclear (sn) RNA that is *trans*-spliced onto all nuclear protein coding genes. The SL provides a 5' cap to the polycistronically transcribed mRNA and has been implicated in mRNA translation [1]. During the investigation of SL RNA biogenesis in *Leishmania tarentolae*, a striking observation was made regarding the transcriptional activity of the SL RNA gene cassette expressed on the pX plasmid [2,3]. Though the copy number of the episomal SL RNA genes was equivalent to the genomic SL RNA genes, the relative transcript levels were vastly different, with the genomic locus contributing ~90% of the total SL [4]. A similar RNA differential was reported in *Leptomonas seymori *[5], although in subsequent studies steady state RNA levels expressed from episomal and chromosomal SL RNA genes were essentially equal [6]. Functionally the relative lower transcript levels in *Leishmania *versus *Leptomonas *have been useful for studying SL RNA gene mutations that would otherwise be dominant negatives at competitive levels. Splicing defective mutations expressed from high copy episomes, presumably due to the relative abundance of the toxic mutated SL RNA, were not tolerated within *Leptomonas *and quickly lost [6].

The SL RNA gene is transcribed by an RNA polymerase (RNAP) II complex [7-11]. As for snRNAs of other eukaryotes, the SL RNA gene is under the control of upstream promoter elements. The promoter recruits transcription factors that are functionally homologous to TBP and the human snRNA activating protein complex (SNAP_c_) [10,12-16] as well as homologs of TFIIA and TFIIB [10,17,18]. The SL RNA genes in *L. tarentolae *are organized into two separate, head-to-tail tandem arrays, *MINA *and *MINB *[19]. The *MINA *array contains ~60 gene copies with a 363-bp repeat length of which 105 are transcribed, resulting in a 96-nt mature transcript. The *MINB *array has ~40 copies, with a periodicity of 296 bp. The transcribed regions in both arrays are identical. The non-transcribed regions contain a bipartite promoter at -67/-58 (the -60 element) and -40/-31 bp (-30 element) from the transcription start site in the *MINA *array [2,4]. The *MINA *tagged SL (tSL) RNA gene cassette in the pX episome contains the transcribed region with an 11-bp mutation in the 28–39-nt region of the exon, 100-bp of upstream sequence including the -60 and -30 elements, and 45 bp of sequence downstream of the transcription-termination inducing T tract [20]. When transfected with the circular pX episome, *L. tarentolae *multimerizes the plasmid into larger circles with five or six complete copies per molecule. The parasite will amplify variably the amount of multimers in any cell, with ratios of genomic:episomal SL RNA genes ranging from 0.5 to 7.4 [4]. The average ratio is ~1.2 [4] and can be influenced by the drug isoform and concentration. Experiments using a four-member, pX-expressed, differentially-tagged, mini-SL RNA gene array containing two full length repeats yielded no significant increase in the transcriptional efficiency of either the *MINA *or *MINB *promoter in the episomal context. Thus the reduction in transcriptional activity is not related directly to the sequence of the SL RNA gene cassette in pX [19].

Transcription in the nucleus tends to occur in discrete sub-nuclear domains [21]. These domains may share the same class of RNAP, but transcribe different classes of genes [11,21]. The variant surface glycoprotein loci in the *Trypanosoma brucei *nucleus migrate to a perinucleolar RNAP I-containing domain upon activation, whereas the distributed rRNA loci congregate at a RNAP I domain in the nucleolus. [22]. Transcription of the SL RNA gene locus in the *Trypanosoma cruzi *epimastigote occurs in a single spot, in this case a perinucleolar RNAP II factory [11].

Template access and nucleosome positioning are vital to RNAP II transcription initiation in eukaryotes [23]. A survey of nucleosome positions in yeast revealed extensive nucleosome-free regions 200 bp immediately before RNAP II transcribed genes [23]. This nucleosome-free region generally includes the upstream activation sequences necessary for transcription initiation. Nucleosome placement on promoter elements at a number of loci in yeast is associated with quiescence [24-29]. Bending of DNA by nucleosome(s) may activate transcription, for example by bringing enhancer-binding proteins into contact with the basal transcription machinery as is the case for the human U6 promoter [30]. Consistently, chromatin repression can be relieved by protein:DNA binding, such as transcription factor:promoter interactions [31]. The binding of proteins like TBP can also induce a bend into DNA [32]. Little is known about the rudimentary epigenetic context of SL RNA gene transcription, including the nature of the association between the genomic array and its nucleosomes. The differences in the transcriptional activity of the SL RNA gene array and the pX episome may arise from epigenetic differences such as nucleosome distribution or nucleotide modifications.

To assess structural differences between the episome and the chromosomal array, we localized the SL RNA gene array and the pX episome relative to one another within the nucleoplasm and mapped the nucleosome positions in both the *L. tarentolae *genomic SL RNA gene array and on the SL RNA gene cassette in the episomes. Both the nucleosomal organization and localization of the episomes are different and discrete from the genomic loci. Clonal episome lines have varying transcriptional activities, suggesting a mechanism of epigenetic heredity. These observations provide a tangible explanation for the observed differences in transcription between the episome and the genomic SL RNA gene array.

## Results

### Episomal and genomic SL RNA gene arrays reside in discrete nucleoplasmic zones

Differential nucleoplasmic localization could be a contributing factor in the reduced transcription of the episomal SL RNA genes. The relative positions of the genomic SL RNA gene arrays and the pX-tSL RNA gene episomes were established by fluorescent *in situ *hybridization (FISH). As the genomic SL RNA genes appear in two distinct arrays in the haploid genome, we would anticipate seeing up to four SL RNA gene signals per nucleus. For the episome, the number of discrete molecules is between 20–30, thus the range of possible distributions is between one to several dozen.

**Figure 1:**
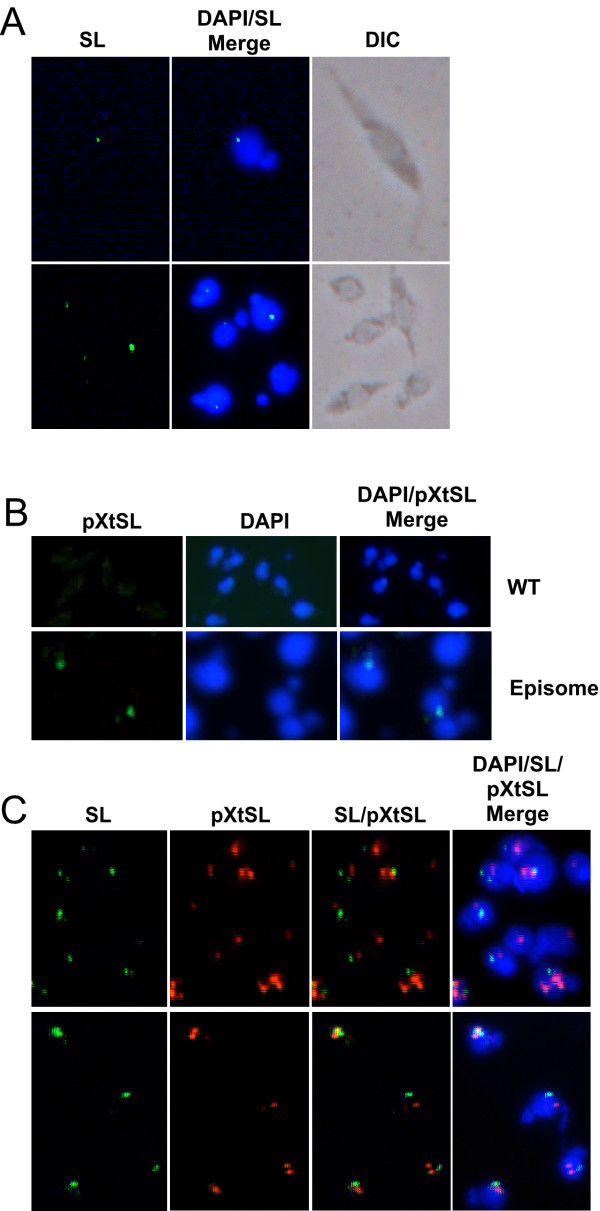
**DNA FISH localization of the SL RNA gene array and pX-tSL episome in the nucleoplasm**. The genomic SL RNA gene array does not co-localize with the pX-tSL episome. A) The genomic SL RNA gene array was visualized using a green Alexafluor 488-conjugated PCR fragment that corresponded to the entire *MINA *SL RNA gene repeat. The genomic arrays reside in a single discrete location within the nucleus. B) Specificity control for the exon-tagged tSL RNA gene. The episome pX-tSL was visualized with an Alexafluor 488-labeled oligonucleotide, NSTAG, in transfected cells. No hybridization was observed to WT cells. C) Double staining for the SL RNA gene array and the episomal tSL RNA genes. The endogenous genes were visualized with an Alexafluor 488-conjugated PCR fragment, and the episome pX-tSL was visualized with a Tamara-labeled NSTAG oligonucleotide probe. There are examples of 1, 2, and three pX-tSL domains within the parasite nucleus.

Initial detection of the SL RNA gene in *L. tarentolae *showed that *MINA *and *MINB *reside in a single spot within the nucleus (Figure 1A). Simultaneous staining with an rDNA nucleolar marker and the SL RNA gene probe showed no consistent positioning of the SL RNA gene array relative to the nucleolus (data not shown). As with *T. cruzi *epimastigotes [11], multiple SL RNA gene spots were occasionally seen (13%) that could be attributed to either background or the process of cell division. Using a probe specific for the tagged portion of the SL RNA gene cassette in the pX plasmid (NSTAG), the episome was localized relative to the chromosomal SL RNA gene locus (Figure 1B,C). Hybridizations with NSTAG in WT *L. tarentolae *showed no nuclear signal, as opposed to pX-tSL transfected parasites, confirming that the probe is specific for the presence of the episomal SL. The episomal genes congregated much like the genomic loci into one or two foci as opposed to being distributed around the nucleoplasm. Immediately apparent was the lack of overlap between the green chromosomal SL RNA gene probe and the red episome probe, indicating that these loci are generally discrete from one another (Figure 1C). Out of 300 cells, the episome localized in two or more spots in 29% of the cells, with two spots being the most prevalent in 24% of the total cells. There was no discernable pattern or orientation to the episome distribution within the nucleoplasm. Co-localization of the episome with the SL RNA gene array was low (3.5%). Though the episome congregates into distinct zones within the nucleus, it does not co-localize with the genomic array.

The low number of foci for the SL RNA genes from either the genomic or episomal templates indicates that the gene sets are physically clustered. While SL RNA gene transcription is maximized from the genomic location, the differential situation of the episomal SL RNA genes could be a reflection of dominance by the transcription machinery involved in the expression of the drug-selected marker gene that is situated on the circular molecule and/or variations in the chromatin at these different locations. To further investigate the differences between the genomic and episomal SL RNA genes, we mapped the nucleosome arrangement at each locus.

### *L. tarentolae *possesses monosomes of standard eukaryotic size

Given the paucity of information concerning transcription initiation and chromatin organization in kinetoplastids, we sought to investigate the chromatin context of both the SL RNA gene locus and pX-tSL RNA gene episome. Nucleosome distributions on the *MINA *and pX-tSL RNA gene templates were mapped by *in situ *micrococcal nuclease (MNase) digestion of chromatin in permeabilized cells. MNase digestion mediates efficient double-stranded breaks in linker DNA and is blocked on nucleosome-associated DNA.

**Figure 2:**
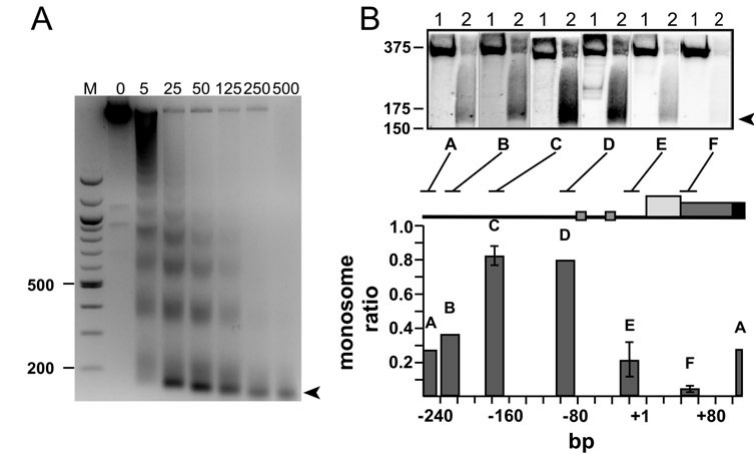
**Detection of nucleosome ladders and positioning on the SL RNA gene repeat**. A) Negative image of an ethidium-bromide stained agarose gel for a typical MNase digestion. Digestion of the *L. tarentolae *chromatin produced a characteristic ladder of DNA fragments. M = 100-bp size marker; 0 = mock treated chromatin. Increasing units of MNase are indicated, ranging from 5 – 500 units/ml. The arrowhead indicates the monosome. B) Southern blot mapping reveals strong preferential protection of an intergenic region between the T tract and the Proximal Sequence Element (PSE). DNA size marker positions are indicated on the left. Lane1, *Sac*II digest of the SL RNA gene in mock-treated DNA; lane 2, MNase digested chromatin. The blot was hybridized with ^32^P-labelled oligonucleotide probes. The letter below each blot refers to the specific oligonucleotide probe, as listed in Table 1. The probe location is indicated by the lines above the schematic. The histogram represents the ratios of the monosome signal: genomic signal.

Digestion of the parasite chromatin with a range of MNase concentrations showed a characteristic ladder of DNA fragments when separated on agarose gels (Figure 2A). The bottom band corresponded to the length of DNA bound by a single nucleosome, or a 'monosome', with higher amounts of enzyme resulting in closer trimming of the bound DNA. The higher molecular weight fragments corresponded in rank order of nucleosomes bound: the second band represents two nucleosomes plus linker DNA, the third band, three nucleosomes plus linker DNA, and so on. From the band distributions on this and acrylamide gels (data not shown), the average amount of DNA bound in a nucleosome was on the order of 160 bp and the linker size was approximately 70 bp.

The nucleosome configuration in *L. tarentolae *by this assay is comparable with those seen in *T. brucei *and *T. cruzi *as well as higher eukaryotes [33,34]. The profiles of the SL RNA gene genomic and episomal arrays were determined by specific probing of the protected DNA.

### The *MINA *array nucleosomes lie on sequences not involved in transcription

By using a selected series of oligonucleotide probes we can determine areas of DNA protection by nucleosome binding [35]. Blotting of the digestion products was first used to discern the state of nucleosome positioning on the genomic array. The probes scan a region of interest and the nucleosome position is inferred from the amount of protection seen in the monosome band. Blots were prepared by electro-blotting samples from a native acrylamide gel that showed better resolution and transfer characteristics than capillary transfer from agarose gels.

Wildtype cells were used for the Southern blot nucleosome mapping analysis of the genomic array. Probes targeting the *MINA *SL RNA gene array (listed in Table 1) revealed regularly positioned nucleosomes that occupied sequences primarily in the intergenic non-transcribed spacer between the T tract and the promoter elements (Figure 2B). Primers that recognize the region between -237 and -92 showed the strongest signal (probes C and D) indicating that this region is bound by a nucleosome. The signal tapered off as the primers move away from these coordinates. Within the SL RNA gene transcribed region (probe F) the sequence was essentially unprotected, indicating an absence of nucleosome association with the transcribed region. Probes A, B, and E hybridized to regions with some protection from MNase digestion: A and B were likely at the 5' edge of possible nucleosome positions within the array, while probe E, situated between -28 and -7, may be revealing protection due to nucleosome occupancy or potential transcription factor binding, as it is located near the promoter elements [4,5,36]. To control for differences in hybridization efficiency, each probe signal was normalized against a uniform digestion product run alongside the nucleosome-protected region. The general pattern of protection was established in multiple replicated experiments. Experimental error was estimated by comparing the ratios between a probe and its reverse complement using the same method for deriving hybridization conditions. The histogram bar represents the average signal ratio between the reverse complemented probes and the error bars show the range between the two ratios.

The protection blots indicated regular patterns of nucleosome placement that are distinct from the average nucleosome distribution seen in total DNA analysis. The MNase-exposed region in the *MINA *array revealed an inter-nucleosomal linker of approximately 180 bp, as opposed to the average 70-bp linker, while the protected DNA fell in the anticipated 160 bp vicinity. Primer F falls in the transcribed region of the SL RNA gene that is common to both *MINA *and *MINB *arrays, neither of which are thus showing any signs of nucleosome protection. The *MINB *array has a smaller repeat size that may preclude the successful placement of a nucleosome between the SL RNA genes. As the distribution within the *MINA *array appeared regular, the borders of nucleosome protection were next mapped using a primer extension protocol.

### The precise position of a nucleosome on the *MINA *SL RNA gene

Primer extension mapping was performed to confirm the blot results and to map precisely the locations of the high occupancy nucleosomes on the *MINA *SL RNA gene array. The probe C primer and its reverse complement were chosen as the extension initiation points due to the high level of protection measured at that location. MNase can produce single-stranded nicks within the nucleosome, although at relatively lower efficiency than double-stranded breaks in linker DNA, and with a higher affinity for digesting A-T rich sequences. Mapping of the SL RNA gene's MNase sensitive sites was achieved by titrating the amount of enzyme in the digestion reaction to find the best conditions for extension analysis of the naked DNA. Typically, the optimal concentration of MNase used to map the nuclease sensitive sites on the naked DNA was ~100 fold less than that used to digest the chromatin *in situ *(data not shown).

**Figure 3:**
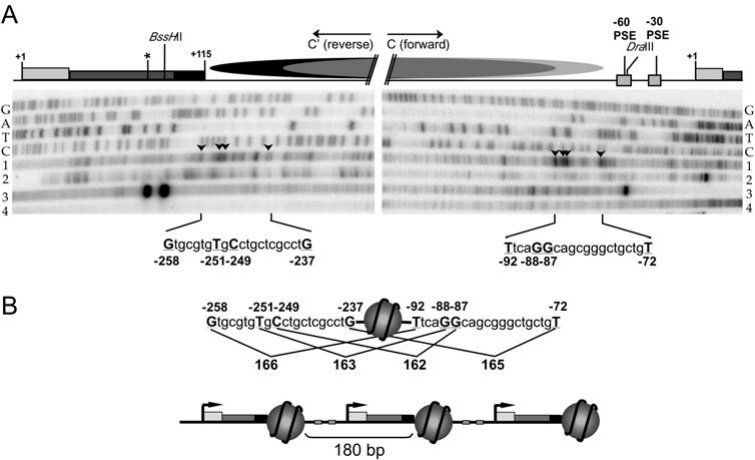
**Primer extension mapping of the nucleosomes on the SL array**. A) The panel on the left represents the 5' end of the nucleosome, and the right panel the 3' end. The schematics along the top edge of the extensions depict the sequence as it relates to the SL RNA gene map. Oligonucleotide C and C' were used to generate the results. The light gray and dark ovals represent total range of nucleosome protection along the sequence. Lane 1, MNase-digested chromatin; lane2, MNase-digested purified DNA; lane 3,*Bss*HII digested or *Dra*III-digested DNA, respectively; lane 4, Mock treated DNA. Arrowheads in lane 1 indicate bands resulting from nucleosome protection. The sequences below the extensions map the major 5' and 3' ends of the protected DNA. *: anomalous *Bss*HII digestion product. B) Schematic of nucleosome organization on the SL RNA gene array. The genomic SL RNA gene array has a regular arrangement of nucleosomes confined to the non-transcribed spacer regions. The top schematic highlights the regular ~164-bp phasing between the major PE stops. Four potential positions of the nucleosome are presented relative to the DNA sequence of the spacer region. The bottom diagram depicts the conformation of the nucleosome in the SL RNA gene array. ~180 bp encompassing the upstream promoter sequences and the transcribed region are free of nucleosome binding.

Primers located in nucleosome-protected fragments were extended and separated on a high-resolution gel alongside a sequencing reaction using the same primer (Figure 3). The arrowheads highlight bands in the MNase-digested chromatin (lane 1) that were unique both in presence and prevalence when compared to the naked, MNase digested DNA (lane 2). Strong bands present in the lane 1 that were not visible in either lane 2 or 4 were interpreted as the ends of the nucleosome-protected regions. Mapping the 5' boundaries of the *MINA *nucleosomes showed four definitive stops at nucleotides -258, -251, -249, and -237 relative to the start site of transcription. The 5'-most band corresponded with the 3'-end of the poly-T tract downstream of the preceding SL RNA gene, the termination signal for the SL RNA gene transcription complex [37]. The primer extension positive controls in lane 3 of each extension showed two major stops for *Bss*HII and one for *Dra*III. The second band seen in the *Bss*HII control was unexpected and may represent sequence heterogeneity within the *MINA *non-transcribed region. However, this unexpected pattern is likely enzyme-mediated, given that all other restriction enzymes used in this assay produced the predicted number of stops, and *Bss*HII is known to exhibit star activity. There were four definitive stops in the 3' map with similar relative spacing to the results for the 5' map. The stops mapped to positions -92, -88, -87, and -72. Taken in pairs, the gaps measured 166 bp, 163 bp, 162 bp, and 165 bp, suggestive of a nucleosome core particle (146 bp)[38] plus histone H1 (~20 bp) protection [39] (Figure 3B).

The extension map was in agreement with the probing results, and extended the protected area to a region between -72 and -258, within which four groups of protection indicated that the prevalent nucleosome core particle or nucleosome core particle plus histone H1 sizes are approximately 164 bp.

### The episomal tSL is obscured by nucleosomes

We continued by probing nucleosome-protected DNA fragments to assess the nucleosomal arrangement on the episomes. In these experiments an additional control lane was included to detect any cross-hybridization between genomic DNA and the episome-specific probes.

**Figure 4:**
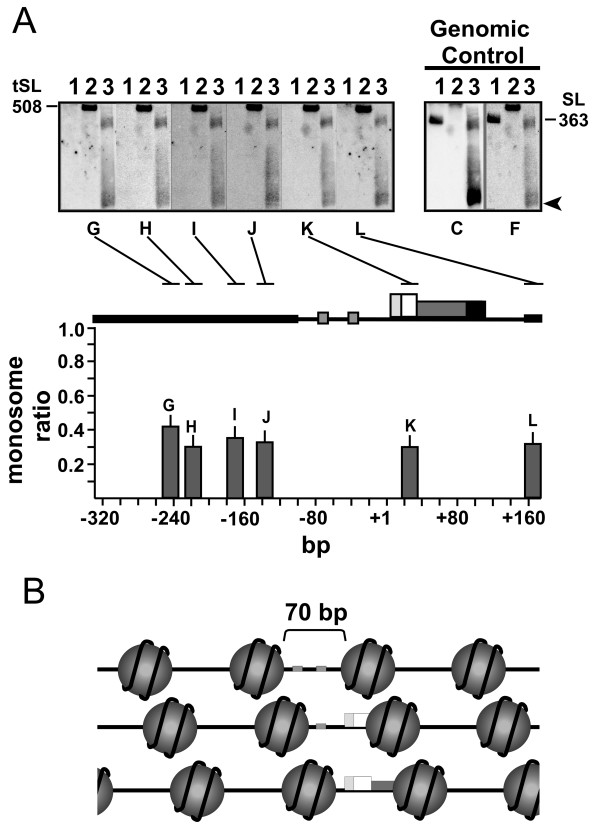
**Southern blot mapping of nucleosomes on the episomal pX-tSL cassette**. A) Unlike the results for the genomic array, the pX-tSL cassette shows no preferential protection. The letter below each blot refers to the specific oligonucleotide hybridization probe, as listed in Table 1. The probe location is indicated by the lines above the schematic. The schematic shows the location of the SL RNA gene, the upstream promoter elements, and the borders of the cassette (thick black lines). The tag sequence is represented as a white box. The histogram represents the ratios of the genomic signal:monosome signal. Lane1, *Sac*II digest of mock-treated DNA; lane 2, *Bst*EII/*Not*I digest of the pX-tSL mock-treated DNA; lane 3, MNase-digested chromatin. B) Three hypothetical episomal nucleosome array conformations, to which only the first is able to bind the SL RNA gene pre-initiation complex. Assuming the periodicity of the nucleosomes on the drug-selectable marker-carrying episome are regular for *L. tarentolae*, the tSL RNA gene promoter has at most a 26% chance of exposure to cognate transcription factors.

Southern blots were probed with a series of oligonucleotide probes (Figure 4). Episome-specific probes G through L hybridized with almost the same signal strength over a 500-bp stretch, including probe K which annealed to the tSL specific tag in the transcribed region. Reverse complement probe estimations of error indicate that the differences between the probes are likely due variance in hybridization conditions. As with the data in Figure 2, the pattern of protection was established on multiple blots. Genomic wildtype DNA negative controls were blank for these probes (lane 1 for each hybridization), validating the origin of the episomal sequence protection. The data from these probes are presented as ratios for direct comparison below the blot. Two of the genomic probes were also used: Probe C was specific for *MINA*, absent from the tSL RNA gene cassette, and showed no signal in the episome-control lane 2, while probe F cross-hybridized with the episome. The hybridization in lane 3 confirmed the protection seen with probe K, and validated the conclusion that the transcribed region was protected from MNase digestion, likely by nucleosomes, in the episomal copies.

Hybridization for the pX-tSL RNA gene showed a different pattern for the episome relative to the genomic array. Whereas the genomic locus had a pattern indicative of positioned nucleosomes along the array, the episomal copies, which in the experimental culture were present at 130% of chromosomal (data not shown), showed no preferential protection. The probability of protection between any of the areas analyzed within the 500-bp region was approximately the same, indicative of nucleosome occupancy across the entire sequence distributed across the population of molecules. The blotting results for the pX-tSL RNA gene episome inidicated randomly distributed nucleosomes within the transfectants. Primer extension mapping was performed as on the chromosomal locus and no informative bands were produced (data not shown), as anticipated for a randomized nucleosome distribution.

### Clonal lines exhibit minor differences in episomal transcript accumulation

One difference between our transfection studies and those performed in *Leptomonas *was the absence of single-cell cloning for the *Leishmania *transfected lines [6]. To explore the effect of cloning we isolated several clones from our pX-tSL RNA gene transfection stock and examined the relative levels of episomal versus genomic SL RNA transcripts and SL RNA genes. All of our previous observations on transcriptional activity of the pX episome used uncloned populations of *L. tarentolae *transfectants. This simple difference could account for the transcriptional disparity between the two systems, and predicts that cloned transfectants have varied transcriptional activities. Uncloned populations of cells expressing green fluorescent protein from epsiomal plasmids show heterogeneous levels of protein expression (data not shown). In an uncloned population there could either be a range of cells that express the tSL RNA gene cassette at different levels that average out to 5–10% of total SL RNA production; alternatively, each cell may express the tSL RNA at the same, relatively low rate.

**Figure 5:**
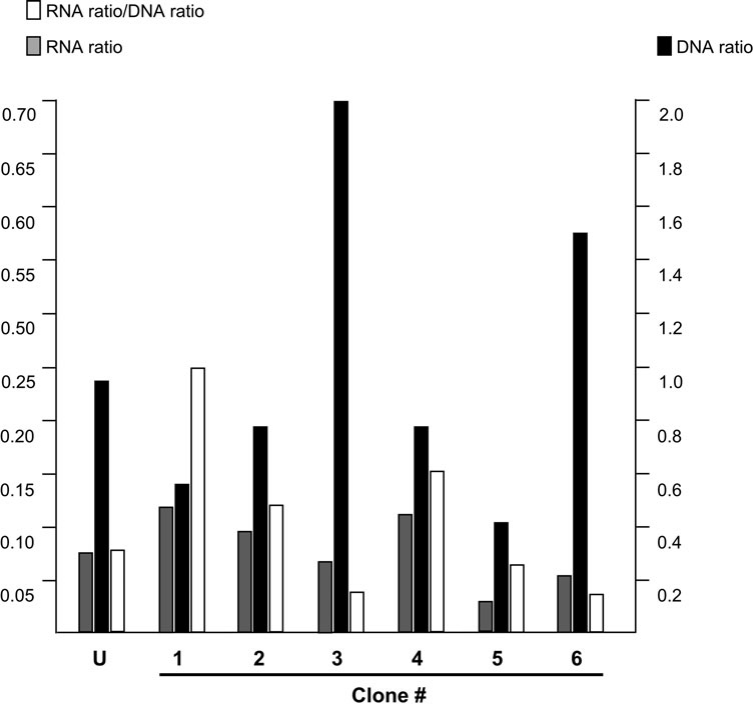
**Clonal variance in tSL RNA gene transcript**. Histogram showing variations in steady state RNA levels and episomal DNA quantities for the uncloned pX-tSL transfectants (U) and the derived clonal lines (1–6). Relative RNA levels were quantified by poisoned primer extension and relative DNA levels were quantified by Southern blotting. The ratio of the episomal tSL RNA transcript to the genomic SL RNA transcript is plotted by the gray bar and the corresponding episomal:chromosomal using the left hand scale. The DNA ratio is plotted by the black bar using the right hand scale. The white bar plots the RNA ratio divided by the DNA ratio using the left hand scale. Assuming factors influencing steady state levels of RNA are equivalent between the all the transfectants, the white bar gives an estimate of the pX-tSL episome transcription activity per gene copy. The pX-tSL clonal lines exhibit different expression rates.

After cloning, the variability of expression and the nature of that variation within the *L. tarentolae *population were examined. Poison-primer extension was used to quantify the ratio of endogenous to tSL RNA expressed in the clonal lines, while Southern blots reported the episome quantity normalized to the genomic copies (Figure 5). A ddATP-poisoned reaction halted polymerization 2 nt from primer F in the endogenous SL RNA versus 8 nt in tSL RNA substrate, creating extension products of 23 and 29 nt, respectively. The six clonal lines and an uncloned control showed substantial variation among tSL RNA levels expressed as a percentage of wildtype SL RNA; none approached parity with the genomic levels (Figure 5, gray bars). An additional consideration in this comparison is the episomal copy number, variation in which could result in a direct dosage effect. The signals were quantified and the amount of pX-tSL RNA gene in each clone was expressed as a ratio of the chromosomal copy (Figure 5, black bars). Comparing the percentage of RNA produced with the copy number of pX-tSL RNA gene, there was no direct correlation with the tSL RNA produced and the normalized amount of episome. By dividing the ratio of RNA expression by the ratio of episomal copy numbers, we derived a metric for the normalized transcriptional activity for each population of pX-tSL RNA gene episomes. Episomal expression showed an eight-fold difference in their per-copy, normalized magnitude from a low of 3.4% (clone 3) to a high of 24.5% the level of wildtype (clone 1) relative to a single copy of the genomic SL RNA gene. Southern blot nucleosome mapping of clone 1, the most actively transcribed clone, showed no substantial differences from the uncloned cells (data not shown).

The transcript accumulation levels per episome copy in each cloned population varied eight-fold, but the level of tSL RNA never rose above 13.5% of the wildtype SL RNA population. In *L. tarentolae *there may be a dynamic process that limits the amount of tSL RNA produced by decreasing the episomal copy number in situations where the transcriptional activity from the episome is too high. The tSL RNA may be toxic at levels great than 13–15% in the parasite, although this SL RNA behaves as wildtype for all intents and purposes [1,20,37]. Similar arguments have been made to explain the inability of some episomal SL RNA gene mutations to be expressed in *Leptomonas *[6]. This dynamic process may be able to reduce the expression from episomes with substandard or toxic SL RNA gene cassettes within limits, however the baseline expression of selectable marker must be maintained such that the cells survive drug pressure.

### The SL RNA gene promoter DNA is bent in association with the pre-initiation complex

Considering the bends induced within a genomic array due to nucleosome association and active transcription initiation, the SL RNA gene itself could exist in a topologically complex structure. Nucleosomal condensation and transcription-initiation complex binding could contribute to the discrete sub-nucleoplasmic localization seen *in situ*. We next turned our attention to how the SL RNA gene pre-initiation complex (PreIC) might affect the topology of the gene. Nucleosomal binding creates a bend in the DNA of 85° [39], likewise the binding of transcription factors to their specific recognition sites is often accompanied by, or enabled by, induced bending of the DNA [30,32,40]. We examined the bending potential of the SL RNA gene PreIC on the -60 element using the pCY7 plasmid [41] containing the -67/-58 sequence cloned between the *Sac*I and *Bgl*II sites.

**Figure 6:**
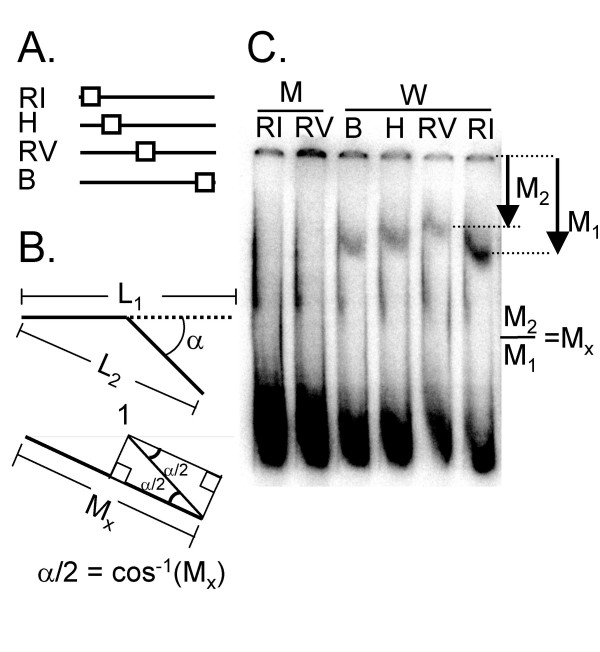
**Binding of the PreIC to the -60 PSE inducesbending of the DNA**. A. Location of the -60 PSE (open box) relative to the 409-bp restriction fragments released from pCY7. B = *Bam*HI; H = *Hin*dIII; RI = *Eco*RI; RV = *Eco*RV. B. Diagram showing the formula used to calculate the bending angle from measured parameters [41,57]. L1 = length of DNA; L2 = end-to-end distance of bent *Eco*RV-digested DNA. M_x _= M2/M1 (Fig 1C) ≅ L2/L1 (Fig 1B). C. EMSA analysis of different pCY7 restriction fragments incubated with 30% ammonium sulphate cut of *L. tarentolae *nuclear extract. M1 = mobility of *Eco*RI digested DNA; M2 = mobility of *Eco*RV digested DNA. W = wild type binding site; M = 10-bp transversion mutation of the binding site.

The -60 element can be released from the plasmid on identical-sized (409 bp) restriction fragments at multiple positions relative to the ends of the DNA (Figure 6A). Induced bending of the DNA at different positions will result in altered mobility of the protein-DNA complex in an electrophoretic mobility shift assay (EMSA) [41] as illustrated (Figure 6B). The ~300 bp restriction fragments were incubated with *L. tarentolae *nuclear extracts and their relative mobility measured. The mobility of the maximally-bent *Eco*RV-released DNA, with the -60 element in the middle, was 73.2% of the mobility in EMSA of the minimally-bent *Bam*HI- and *Eco*RI-released fragments where the -60 element was located closer to either end (Figure 6C) meaning that the maximally-bent fragments possessed a relative mobility (M_x_) of 0.732. Using the formula illustrated in Figure 6B the DNA flexure angle induced by the -60 PreIC was calculated to be 82° ± 4° from an average of three experiments.

In all experiments, binding of the complex to the mutated probes was never observed (Figure 6C), thus we conclude that a component of the -60 PreIC plays a distinct role at the SL RNA gene -60 element by inducing a bend. An induced bend in the SL RNA gene promoter affects the topology of the array and could have differential effects on transcription of genomic versus episomal copies of the genes.

## Discussion

Here we examined the spatial and structural organization of genomic and episomal versions of SL RNA genes to assess epigenetic factors that could affect levels of gene transcription. The episome and genomic arrays do not co-localize within the parasite nucleus. The chromatin in *L. tarentolae *follows a regular pattern of approximately 160-bp nucleosome-bound DNA separated by an approximately 70-bp linker region. The nucleosomes on the genomic SL RNA gene array, however, are essentially excluded from the 185-bp promoter and transcribed regions, and reside in a narrow range of positions within the non-transcribed intergenic spacer. In addition to nucleosome-induced bending, transcription factor binding at the SL RNA gene -60 promoter element may introduce a further 80° kink into each array repeat. In contrast with this transcriptionally-permissive arrangement in the genomic array, nucleosomes on the episomes are randomly distributed over the tSL RNA gene cassette, potentially obscuring the tSL RNA gene promoter up to 74% of the time. Cloning of the pX-tSL RNA gene transfectants had a notable effect on the tSL RNA gene transcript contribution of the episome, but did not approach genomic levels nor show significant alteration in nucleosome distribution. The combination of differential sub-nucleoplasmic localization and nucleosome arrangements between the genomic and episomal SL RNA genes indicate that altered access to the SL RNA gene promoter elements accounts in large part for the variance in SL RNA relative to tSL RNA. The SL RNA genomic nucleosome placement is consistent with RNAP II promoter architecture in other organisms, such as yeast [23,42]. Given the similarities between the SL RNA gene transcription initiation complex, what is known about RNAP II initiation, and the nucleosome position relative to the PSE, we infer that efficient binding of the PSE by the initiation complex cannot occur while the PSE is bound in a nucleosome.

Factors other than chromatin configuration can contribute to epigenetic effects on transcriptional regulation. Such factors include DNA modification [43] and topology [44]. DNA in the African trypanosome does not contain the typical modifications (e.g. m^6^C) found in other eukaryotes [45]; the main modified base found in kinetoplastids, β-D-glucosyl-hydroxymethyluracil, is referred to as base J [46]. As well as its association with inactive VSG expression sites in bloodstream form *T. brucei*, base J is associated with repeated sequences including the SL RNA gene array [46]. Base J is thus a candidate for investigation in differential gene expression. Base J is found in *Leishmania *[47], however we have not tested for its presence in *L. tarentolae *SL RNA genes in either the chromosomal or episomal context.

The *T. cruzi *SL RNA gene array resides in a specialized RNAP II transcriptional domain [11]. The *L. tarentolae *SL RNA genes are likely to reside in a similar specialized domain given the chromosomal arrays localization in a single nuclear spot. In *L. tarentolae*, the probe used to localize the arrays did not distinguish between the *MINA *and *MINB *loci. A single spot in the nucleus argues in favor of a single specialized transcription zone for SL RNA gene expression. Likewise, the discrete localization of the episomes are consistent with the concept of transcriptional zoning. The episomes reside as a series of 20–30 concatamerized circular DNA species within the nucleus. In *Leptomonas *and *L. major*, catenated episomes have been observed, but the steady state number of these species are relatively small compared to the free circles [48,49], thus interlinkage is likely unrelated to the observed nucleoplasmic aggregation. Pressure to remain grouped within discrete nucleoplasmic zones may be due to transcriptional optimization of the selectable drug marker. The action of the episomal processive mRNA complex is vital to cell survival, while the activity of the tSL RNA gene transcription complex is incidental.

The bend angle of 82° measured for the *L. tarentolae *SL RNA gene promoter is similar to those measured for TATA-box promoter-bound TBP in humans [32]. At present, we cannot attribute this bending to either *Lt*TBP or some other factor in the *L. tarentolae *PreIC. Bending of DNA upstream of the vertebrate U6 snRNA promoter is mediated not by TBP and SNAP_c _but by a precisely-positioned nucleosome [30]. Since the *L. tarentolae *-60 element is essential for transcription [2,50], alterations in the local DNA conformation may act to facilitate transcription initiation by inducing higher-order structure. The SL RNA genes of *T. cruzi *are adjacent to the nucleolus in transcriptionally-active cells, yet dispersed in the nucleoplasm with the presence of transcription inhibitors [11]. A regularly-spaced bend that occurs in a tandem SL RNA gene array could induce a solenoid-like superstructure, at one extreme. Such higher order structure is consistent with the compact nature of actively-transcribed SL RNA genes in *T. cruzi*. The cumulative effects of inherent DNA bending by the nucleosome [39] and by the PreIC binding could further affect template availability.

Considering only nucleosome placement, we predict that the 10-bp SL RNA gene -60 element will be in an open conformation 26% of the time. Our experimental observations of SL RNA gene transcription from the episome average ~10% the activity of the genomic SL RNA gene, thus the -60 element being in an open conformation is likely not the sole determinant of transcription initiation. The model assumes that all open conformations are equivalent, however this is an oversimplification since subsequent recruitment of other transacting factors or the loading of RNAP II onto the template may require a nucleosome free region downstream of the -60 element. Additionally, the estimated size of the *L. tarentolae *PreIC (Stokes radius: 38.5 Å [16]) is significantly larger than the nucleosome (coiled radius: 21 Å [38]). The steric interactions between the nucleosome and the PreIC may preclude efficient binding to the -60 element for a number of different conformations. Physical separation of the episome from the site of SL RNA gene transcription may negatively impact efficient transcription. Factors that mediate SL RNA gene transcription may be concentrated within an SL RNA gene transcription 'factory' and hence sequestered from the episome. Finally the activity of the processive mRNA RNAP II complex may interfere or compete with the SL RNA gene RNAP II complex initiation. In pX-tSL RNA gene episomes a stable 'runaround' transcript of ~1400 nt containing the tSL RNA gene sequence accumulates [37]. The transcript is not produced by the SL RNAP II complex nor does it recognize the transcription termination signal for the SL RNA gene. The processive transcription may serve to physically disrupt the SL RNA gene PreIC and thus further hinder transcription initiation. As in the CHA1 locus in yeast [24], transcription of the processive complex might disrupt and moderately scramble the nucleosome positions through the region, serving to occasionally push the nucleosomal arrays into an active or inactive state *vis à vis *SL RNA gene transcription. The processive complex disruption of the nucleosomes could also act as a positive modifier of SL RNA gene transcription initiation, as the disruption of nucleosomes by the processive complex, perhaps by polymerase-associated nucleosome disassembly complexes similar to FACT [51] may increase the chances for the initiation complex to bind to a previously nucleosome occluded -60 region.

We have correlated the repression of episomal transcription with nucleosome association. The specific periodicity of the nucleosomes within the *MINA *array is distinct from that of the average nucleosomal array in *L. tarentolae*, and may reflect the dynamics of transcription factor association, active transcription, and nucleosome placement in non-transcribed regions. Because the intergenic regions in the array are not transcribed [2], nucleosome placement may be passive, i.e. the activity of the SL RNA gene transcription factors intrinsically excludes nucleosomes from the transcribed regions and by default, the nucleosomes can only associate with the non-transcribed intergenic sequences. The transcriptional control elements present on the pX-tSL RNA gene episome, specifically the T tract, retain function in the probable absence of the downstream nucleosome found in the genomic array [37]. Alternatively, the regular spacing may be actively established and maintained by a chromatin-remodeling factor. The examination of nucleosome placement within other highly-transcribed arrays such as the ribosomal genes will clarify the role of nucleosomes as transcriptional regulators. Speculating on the arrangement of nucleosomes in the 1.4-kb *T. brucei *SL RNA gene array, which encodes a ~150-nt primary transcript and has the largest non-transcribed intergenic region characterized among the kinetoplastids, we would envisage 220-bp nucleosome-free regions spanning the promoter element and transcribed regions separated by up to five bound nucleosomes. Likewise, SL RNA gene arrays that are interspersed with 5S rRNA genes [52] may limit nucleosome binding to the non-transcribed regions, space permitting. The length of the *MINA *array is just sufficient to accommodate a single nucleosome, a fortuitous situation for our nucleosome mapping.

## Conclusion

We describe two epigenetic features for episomal and genomic SL RNA genes that may result in lower expression of the episomal cassette. In *L. tarentolae *the SL RNA genomic and pX-tSL RNA gene episomes reside in discrete sub-nuclear locations. Nuclease protections indicate that a single nucleosome is positioned on the chromosomal SL RNA gene array in the intergenic region, leaving the promoter and transcribed gene region nucleosome-free. The array periodicity of one nucleosome per 363 bp differed from the standard heterochromatin arrangement in *L. tarentolae *of one nucleosome per 230 bp. The array is bent further by the interaction with transcription factors. By contrast, nuclease protection studies suggest that the pX-tSL RNA gene episome nucleosomes were distributed evenly. Nucleosome arrangement may be vital for efficient transcription initiation. We estimate the maximum percentage of promoter accessibility at 26% in the episomes, and other competing factors explain the observed episomal transcription levels of 10% in *L. tarentolae*. The presence of distinctly different transcriptional activities in cloned pX-tSL RNA gene lines argues for maintained epigenetic context for each clone.

## Methods

### Cell culture and episome constructions

The *L. tarentolae *UC(A) strain parasites were used for all subsequent assays and transfections. Cells were grown in Brain Heart Infusion media supplemented with 100 mg/ml of hemin at 28°C. Transfections were performed as described [2]. The episome construct, pX-tSL, contained a 250-bp fragment of the *L. tarentolae *SL RNA gene locus with a mutated region in bases +28 to +39 that served as a molecular tag, tSL. Transfectants were selected and maintained in 200 μg/ml of paromomycin (PGC Scientific). Cell lines were cloned on paromomycin/BHI agar plates following a protocol provided by A. Simpson and L. Simpson [53].

### DNA-DNA fluorescent *in situ *hybridization

The *L. tarentolae *cells were grown to mid log phase (3–7 × 10^7^) and fixed in 2% formaldehyde/1× PBS for 15–30 min. Fixed cells were washed twice, resuspended in PBS, and adhered to slides coated with 0.5 mg/ml poly-L-lysine (Sigma). The cells were permeabilized with 0.1% NP-40/1× PBS for 10 min. The slides were washed three times with PBS, and incubated with 10 μg/ml proteinase K (Roche), 10 μg/ml RNase A (MP biomedical), 1× PBS at 37°C for 10 min. The treated slides were washed three times in 1× PBS and dehydrated with sequential, 2 min washes in cold 70%, 90%, and 100% ethanol, and allowed to dry. The DNA was denatured prior to hybridization at 85°C in 70% formamide, 2× SSC for 5 min. Hybridizations and washes were the same for both oligonucleotide and PCR prepared probes. Hybridizations were performed in 50% formamide, 1× SSPE, 10% Dextran, 250 μg/ml salmon sperm DNA. PCR prepared probes were used at a concentration of 3 μg/ml, oligonucleotide probes were used at 1 μg/ml and the hybridization/probe mixture was denatured at 85°C for 10 min, spun down and plunged into an ice bath. The denaturing solution was removed from the slides and the predenatured probe/hybridization solution was applied. The slides were then incubated at 85°C for 3 min and transferred to a humid chamber prewarmed to 60°C. The chamber was equilibrated to 35°C and the slides incubated overnight. The slides were washed twice in excess 50% formamide, 2× SSC at 35°C for 15 min. The slides were then washed twice in excess 2× SSC at 50°C for 15 min. The final wash was performed in 1× SSC at room temperature for 15 min. The slides were dried and mounted with Vectashield mounting medium (Vector labs) containing 70 μg/ml 4',6-diamidino-2-phenylindole (DAPI; Sigma). Images were visualized using either a 100× or 63× objective on a Zeiss Axioscop2. Images were captured with a Zeiss Axiocam using the Axiovision software suite. (Carl Zeiss Inc.)

### Fluorescent probe preparation

Two types of DNA probe were used: oligonucleotides synthesized with incorporated fluorophores, or PCR-amplified dsDNA fragments that had fluorophores subsequently conjugated to guanine residues. The PCR probes were labeled with the Ulysis Alexafluor 488 (green) (Invitrogen). Amplified probes were purified from the initial PCR reaction using DNA purification columns (Qiagen) and either restriction digested or DNase I digested down to fragments of ~100–200 bp (Invitrogen).

### Microccocal nuclease digestion

Fifty milliliters of *L. tarentolae *cells were grown to mid log phase (3–7 × 10^7 ^cells/ml) and centrifuged at 3500 g for 5 min. The cell pellet was resuspended and washed twice in chilled incomplete MNase Protection Assay (MPA) buffer (20 mM HEPES pH 8.8, 10 mM potassium glutamate, 150 mM sucrose, 2.5 mM calcium chloride). The cells were then permeabilized by treating with 500 μg/ml lysolethicin in completed MPA buffer (completed with the addition of 1 mM DTT and 10 μg/ml leupeptin) for 10 min. The cells were washed twice and resuspended at 2 × 10^9 ^cells/ml in complete MPA buffer. Microccocal nuclease (USB) was added to 200 μl aliquots of cells. The MNase was titrated over a range of 5–1000 units/ml. Cells were incubated at 37°C for 3 min, the reactions were stopped, and the cells lysed with the addition of 200 μl of 2× Stop solution (10 mM EGTA, 2% SDS, 500 μg/ml Proteinase K (Roche)). The lysates were incubated at 55°C for 4–16 h. These digestions were then phenol/chloroform extracted, precipitated with the addition of 1/10th volume of 3 M sodium acetate pH 5.0 and 2–3 volumes of ethanol, and incubated at -20°C for a minimum of 2 h prior to centrifugation. The prepared DNA was resuspended in TE and treated with 100 μg/ml RnaseA at 37°C for 1 h prior to further analysis.

### Southern blot nucleosome mapping

The mapping protocol was adapted from Gregory and Hörz [35]. The MNase digested samples along with restriction-digested, mock-treated DNA were separated on 0.5× TBE/8% polyacrylamide native gels. Equivalent cell volumes were loaded for all mock-treated and MNase treated DNA. The DNA was transferred onto nylon membranes (GE Bioscience) as described [54]. Membranes were UV cross-linked (Stratagene), and probed with 5'-end labeled oligonucleotides. The 5'-end labeling was performed using T4 polynucleotide kinase (New England Biolabs) and γP^32^ATP. The hybridization conditions were as follows: Membranes were pre-incubated in a solution of 6× SSC, 1× Denhardts solution, 0.5% SDS and 10 μg/ml yeast tRNA (Sigma) at 42°C for 10 min-1 h. Twenty-five nanograms of probe were hybridized in 6× SSC, 1× Denhardts solution, 0.1% SDS, and 10 μg/ml yeast tRNA at 42°C for a minimum of 2 h. The blots were washed of excess probe in 0.1% SDS, 2× SSC at a salt adjusted T_m _calculated using the Oligonucleotide Properties Calculator [55]. The wash was performed twice for 30 min. The blots were stripped by incubating the membranes in 0.1% SDS at 85°C, checked for residual signal and then reprobed. The membranes were exposed for a minimum of 2 h on a PhosphorImager screen (GE Bioscience) and imaged on a BioRAD FX Pro scanner (BioRAD). Nucleosome positioning was determined by comparing the signal strength of the monosome with the restriction digested mock treated control. The monosome signal strength was quantified using the Quantity One densitometry suite (BioRAD). Total counts were determined for identical areas over both the monosome and the control band. The comparison was expressed as the ratio of the monosome count to the control count. Experimental error was estimated by reprobing the Southern blots with the reverse complement of selected probes using the same hybridization methodology as above.

### Primer-extension nucleosome mapping

The primer extension protocol was adapted from Ryan *et al*. [56]. High resolution mapping of the MNase digested chromatin was performed using radio labeled primers extended with *Taq *polymerase (CLP). Four to eight μl of the sample and control DNA was added along with 10 ng of the 5'-end labeled, specific primer to a 20 μl *Taq *reaction. The reaction was cycled 20–30 times in a thermocycler (MJ Research) to extend the primers to the end of the MNase-digested template. After cycling, 8 μl of sequencing stop solution (0.05% bromophenol blue, 0.05% xylene cyanol, 20 mM EDTA prepared in formamide) was added to the reaction. The reaction was denatured at 85°C for 5 min and quickly cooled to 4°C in a thermocycler. The extension products were separated on a 1× TBE/6% polyacrylamide/8 M urea sequencing gel (Whatman/Biometra). The gel was transferred to a 0.4 mm Whatman paper, covered in plastic film, and vacuum dried. The dried gel was exposed overnight on an imager screen (GE Bioscience) and imaged on a PhospoImager 445 SI scanner (GE Bioscience).

### DNA flexure angle calculation

*L. tarentolae *nuclear extracts and EMSA were performed as described [16]. The angle of DNA bending by the -60 PreIC was determined as described [41,57]. A 34-bp fragment of DNA containing the SL RNA gene -60 element was cloned into the pCY7 vector [41]. The resulting plasmid contained pairs of staggered restriction sites such that an array of digests yielded fragments of exactly the same size (409 bp), with the -60 element located different distances from the end of the fragment (Figure 5A). Prentki *et al*. showed that when a molecule (of length L_1_) is bent at its midpoint, as would occur during an *Eco*RV digest (yielding an end-to-end length of L_2_), the ratio of end-to-end distances (L_2_/L_1 _in Figure 5B) is approximately equal to the relative mobility, M_x_, of that molecule through a gel [41]. M_x _was calculated by dividing the mobility of the maximally-bent DNA *Eco*RV digested DNA by the mobility of the minimally bent *Eco*RI digested DNA (M_2_/M_1 _in Figure 5C). As described by Thompson et al. [41,57], the angle of the bend was calculated by α/2 = cos^-1^(M_x_).

### tSL RNA quantification

RNA from each cell line was extracted using TriZOL (Invitrogen) as described [58]. Primer extensions were performed as described in [59] with the substitution of a poisoned deoxyadenosine nucleotide mixture in the reaction (200 μM dTTP, dGTP, dCTP (Sigma), and ddATP (GE Bioscience)). The reaction products were separated, treated and visualized the same as the primer extension mapping products described above. Quantification of the extension products was performed using the Quantity One densitometry suite (BioRAD).

### Episome quantification

Genomic DNA from the different clones and controls was isolated with DNAzol (Invitrogen) using the standard protocol. The DNA was digested with *Dra*III and *Not*I (New England Biolabs) restriction enzymes, yielding a *Dra*III genomic fragment of 363 bp and a 220 bp episomal *Dra*III/*Not*I fragment. The digests were separated on a 1.5% low melt agarose gel (Fisher) and blotted onto nylon membranes (GE Bioscience) by capillary transfer with 10× SSC for 4 h. Blots were probed with end labeled oligos with sequences shared by both the episomal and genomic SL RNA genes. Imaging and quantification were performed in a similar manner to the previously described Southern blot nucleosome mapping. The amount of episome was expressed as a ratio of episomal signal divided by genomic signal.

## Authors' contributions

RH performed the localization and nucleosome experiments, data analysis and interpretation, and drafted the manuscript. ST performed the DNA bending analysis. DC conceived of the project and participated in data analysis and interpretation. NS participated in data analysis and interpretation, and drafted the manuscript.

## Figures and Tables

**Table 1:** Oligonucleotides used in this study

Letter	Sequence (5'-3')	Target
**Blot hybridization and primer extension**		
A	TTTTTTTGTGCGTGTG	MINA SL
B	GCCTGCTCGCCGAGGTGTGG	"
C*	CGCGTGCTTGTGCTGGTGTGCG	"
D	CAGCGGGGCTGCTGTGTGGT	"
E	GCCCGGACATGATGGGCGGTGTGTG	"
F	GTTCCGGAAGTTTCGCATAC	"
G	TCTCTCTCTCTCTCACCATC	pX-tSL
H	CATCATCAACTGTCTCTTGT	"
I	TTTCAAGGCTTCCCGAACGC	"
J	GCGTGTGTTGAGCCGTCCACCGTA	"
K	ACTTCCTCGAGGCTGAA	"
L	ATCCTCTAGAGCGGCCGCCA	"
**Revere complement probes**		
C'*	CGCACACCAGCACAAGCACAAGCACGCG	MINA SL
E'	CACACACCGCCCATCATGTCCGGGC	"
F'	GTATGCGAAACTTCCGGAAC	"
G'	GATGGTGAGAGAGAGAGAGA	pX-tSL
H'	ACAAGAGACAGTTGATGATG	"
K'	TTCAGCCTCGAGGAAGT	"
**FISH**		
U3	CCTCCTGCCGTCAATCGAAACGCCT	U3 snoRNA
RRNA	AGCCAGCAGGCACGCCAGTGAGGCATGTCTGG	rDNA IGS
NSTAG	TCCGGAAGTTTCGCATACTTCCTCGAGGCTGAA	pX-tSL

*: denotes oligonucleotides used for primer extension mapping.

Underlined bases represent Tamara fluorophore labels.
